# Supplementary material for: Exploring Maternal Socio-Demographic Factors Shaping Children’s Dietary Patterns in Brazil: Results from the 2019 National Health Survey
Source: Int J Environ Res Public Health. 2024 Jul 29;21(8):992. doi: 10.3390/ijerph21080992 (PMC11353673; doi:10.3390/ijerph21080992)
Supplement: Supplementary file 1 [file ijerph-21-00992-s001.zip › ijerph-3052807-supplementary.pdf]

Table S1. PNS 2019 - Questionnaire - Module L

Módulo L - Crianças com menos de dois anos de idade						
**Em caso de mais de uma criança nascida entre 28 de julho de 2017 e 27 de julho de 2019, os quesitos L17 a L40 referem-se a criança mais nova**						
488	1	L01701	L17	Leite materno?	1	Sim
					2	Não
					9	Ignorado
						Não aplicável
489	1	L01702	L17	Outro leite ou derivados de leite?	1	Sim
					2	Não
					9	Ignorado
						Não aplicável
490	1	L01703	L17	Água?	1	Sim
					2	Não
					9	Ignorado
						Não aplicável
491	1	L01704	L17	Chá?	1	Sim
					2	Não
					9	Ignorado
						Não aplicável
492	1	L01705	L17	Mingau?	1	Sim
					2	Não
					9	Ignorado
						Não aplicável
					1	Sim

493	1	L01706	L17	Frutas ou suco natural de frutas?	2	Não
					9	Ignorado
						Não aplicável
494	1	L01707	L17	Sucos artificiais?	1	Sim
					2	Não
					9	Ignorado
						Não aplicável
495	1	L01708	L17	Verduras/legumes?	1	Sim
					2	Não
					9	Ignorado
						Não aplicável
496	1	L01709	L17	Feijão ou outras leguminosas (lentilha, ervilha etc)?	1	Sim
					2	Não
					9	Ignorado
						Não aplicável
497	1	L01710	L17	Carnes ou ovos?	1	Sim
					2	Não
					9	Ignorado
						Não aplicável
498	1	L01711	L17	Batata e outros tubérculos e raízes (batata doce, mandioca)?	1	Sim
					2	Não
					9	Ignorado
						Não aplicável
499	1	L01712	L17	Cereais e derivados (arroz, pão, cereal, macarrão,	1	Sim
					2	Não

				farinha, etc)?	9	Ignorado
						Não aplicável
500	1	L01713	L17	Biscoitos ou bolachas ou bolo?	1	Sim
					2	Não
					9	Ignorado
						Não aplicável
501	1	L01714	L17	Doces, balas ou outros alimentos com açúcar?	1	Sim
					2	Não
					9	Ignorado
						Não aplicável
502	1	L01715	L17	Refrigerantes?	1	Sim
					2	Não
					9	Ignorado
						Não aplicável
503	1	L01716	L17	Outros?	1	Sim
					2	Não
					9	Ignorado
						Não aplicável
